# Methodologies for Generating Brain Organoids to Model Viral Pathogenesis in the CNS

**DOI:** 10.3390/pathogens10111510

**Published:** 2021-11-19

**Authors:** Hannah K. Hopkins, Elizabeth M. Traverse, Kelli L. Barr

**Affiliations:** Center for Global Health and Infectious Disease Research, University of South Florida, Tampa, FL 33612, USA; hannahhopkins@usf.edu (H.K.H.); emtraverse@usf.edu (E.M.T.)

**Keywords:** organoids, brain, pathogenesis, virus, brain organoid generation, brain organoid models

## Abstract

(1) Background: The human brain is of interest in viral research because it is often the target of viruses. Neurological infections can result in consequences in the CNS, which can result in death or lifelong sequelae. Organoids modeling the CNS are notable because they are derived from stem cells that differentiate into specific brain cells such as neural progenitors, neurons, astrocytes, and glial cells. Numerous protocols have been developed for the generation of CNS organoids, and our goal was to describe the various CNS organoid models available for viral pathogenesis research to serve as a guide to determine which protocol might be appropriate based on research goal, timeframe, and budget. (2) Methods: Articles for this review were found in Pubmed, Scopus and EMBASE. The search terms used were “brain + organoid” and “CNS + organoid” (3) Results: There are two main methods for organoid generation, and the length of time for organoid generation varied from 28 days to over 2 months. The costs for generating a population of organoids ranged from USD 1000 to 5000. (4) Conclusions: There are numerous methods for generating organoids representing multiple regions of the brain, with several types of modifications for fine-tuning the model to a researcher’s specifications. Organoid models of the CNS can serve as a platform for characterization and mechanistic studies that can reduce or eliminate the use of animals, especially for viruses that only cause disease in the human CNS.

## 1. Introduction

The human brain is of interest in viral research because it is often the direct or indirect target of viruses, and many viral families have neurotropic viruses [1,2]. Neurological infections can result in consequences to the CNS such as inflammation (encephalitis and myelitis), neurologic disorders such as Guillain–Barré syndrome, Bell’s palsy and parkinsonism, all of which can result in death or lifelong sequelae [3,4,5,6,7,8]. This is of particular concern because COVID-19 and many other emerging viruses are neurotropic, with the potential to affect much of the human population, and appropriate study models, such as stem cell based models, are still being developed [7,9,10,11]. 

There are a variety of ways to model and study viral disease in the CNS. In vitro assays utilizing immortalized or primary cell lines are useful for mechanistic assays but do not reflect the contributions of other cell types present in the organ, which can limit findings. Animal models used for viral pathogenesis research in the CNS include rodent, non-human primates, rabbit, goats and sheep [12,13,14,15,16,17]. One drawback of using an animal model is the host-range restrictions of viruses such that many animal models lack the necessary receptors for viral entry and attachment [17,18]. Another limitation of using animal models to study viruses is the difference in immunopathology, which has been noted in models such as knockout and transgenic mice [17]. Other limitations of using animal models are the husbandry, time involved, cost, and ethics [17,19,20]. 

An emerging alternative to animal models is 3D cell culture and organoids derived from human stem cells. Brain organoids are three-dimensional cellular structures that self-organize into a structure similar to the human fetal brain and are typically derived from either human embryonic stem cells (hESCs) or human induced pluripotent stem cells (hiPSCs), broadly known as human pluripotent stem cells (hPSCs) [21]. Two research groups developed the earliest methodologies for generating CNS organoids. The Knoblich research group based their method for creating brain organoids on protocols for other types of organoids such as gut and optic cup 3D culture [22,23,24,25]. This method, commonly referred to as the Lancaster method, for generating cerebral organoids (COs), is highly reproducible and has become the basis for the majority of CO generation protocols for disease modeling. The Sasai group led the way for guided brain organoid methodologies by using small molecules to dictate cell fate [26]. 

Organoids can be used to model organogenesis, developmental disorders, and the pathogenesis of viruses in organ systems of diseases difficult to model in animals [21]. The advantages of using an organoid model are that they better reflect human gene expression and development, are more accessible and accurately reflect human biology than animals or immortalized cells [27]. Some drawbacks of using organoids are the isolated nature of organoids, as they lack a functional immune system and vascularization [27]. However, the field is still developing, and more complex models are continuously being engineered [11,27].

Organoids modeling the CNS are notable because they are derived from stem cells that differentiate into specific brain cell types such as neural progenitors, neurons, astrocytes and glial cells [21]. They are different from other three-dimensional cultures, because stem cells self-organize and differentiate into the appropriate cell types to accurately model the human fetal brain [21,28,29]. As a result of this, organoids are also a model for studying gliogenesis and neuronal formation and networking [29]. Brain organoids can be cultured for more than 1 year and can model the post-natal brain when in culture for greater than 250 days [29,30]. 

The typical process of generating a brain organoid starts with hiPSCs or hESC colonies that are separated into single cells, and then are aggregated to form embryoid bodies (EBs), which are typically embedded in Matrigel to provide a scaffold for the growing organoid. EBs are three-dimensional pluripotent stem cell (PSC) aggregates to which growth factors are then added to their media to promote the growth of neuroectoderm, which matures into neuroepithelium and then into cerebral tissue [31].

**Figure 1:**
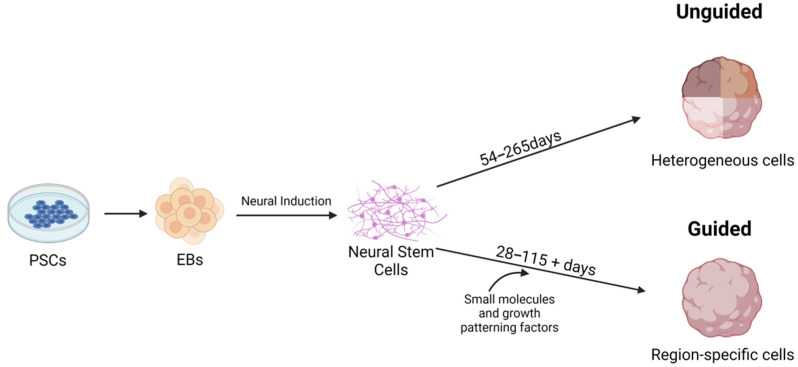
Overview of generating CNS organoids and the differences between guided and unguided methodologies. Figure was created with BioRender.com (accessed on 29 October 2021).

Numerous protocols have been developed for the generation of CNS organoids, and most protocols start with iPSCs that are aggregated into EBs (Figure 1). EBs are induced to form neural stem cells by the addition of neural induction media, which contains factors to inhibit the BMP/TGF-β signaling pathway [29]. Common strategies include SMAD (Suppressor of Mothers Against Decapentaplegic) inhibition and *Wnt* pathway activation [32,33,34,35,36]. SMADs are a family of proteins that are involved with TGFβ signaling [37]. In iPSCs, SMAD inhibition can be achieved through the use of inhibitors such as Noggin and SB431542 [38]. Neural stem cells are self-renewing and generate glia and neurons during embryonic development [39].

There are two main methods for brain organoid generation. The first is to create unguided organoids that utilize iPSC propensity for spontaneous morphogenesis and intrinsic cell signaling [24,29,40,41]. These cells can potentially develop into dorsal forebrain, ventral forebrain, midbrain, choroid plexus, hippocampus, retina, and hindbrain cell lineages [24,29,40,41]. These models are advantageous since they create a heterogeneous population of cells within the organoids [21,29]. These types of organoids, specifically COs, are sometimes called whole brain organoids since they spontaneously model the diverse neural population of the developing brain [25,29]. The main and immediate drawback of utilizing this method is that spontaneous differentiation can lead to unpredictable proportions and arrangements of cells within the organoid, which can be counterproductive for researchers trying to model specific regions of the brain [21,29,40,42]. 

The second method for brain organoid generation is a guided approach in which patterning factors are used to induce specific cell lineages at specific locations within the organoid [21,29]. In guided models, growth factors are applied to developing organoids, and these vary according to the goal of organoid generation according to a specific pattern [21,29]. These patterns can recreate regions similar in structure and cell composition to the cerebral cortex, midbrain, optic cup, choroid plexus, hypothalamus, cerebellum, ganglionic eminences, thalamus, and hippocampus [22,29,34,35,43,44,45,46,47,48,49,50,51,52,53].

Both guided and unguided protocols vary greatly in length of time for brain organoid generation, with the shortest protocol in the literature review being 28 days, and the longest protocols being nearly 2 months [24,25,32,54]. The length of time required is influenced by factors such as brain cell types desired and methodology. The costs for generating a population of brain organoids ranged from just over USD 1000 to about 5000. Costs were calculated by summing the costs of 1 unit of each reagent listed in the protocol. 

Here, we describe the various CNS organoid models available for viral pathogenesis research to serve as a guide to determine which protocol might be appropriate based on research goal, timeframe, experience, and budget. The literature for this review was originally sourced from Pubmed, Scopus, and EMBASE using the search terms “brain + organoids,” and “CNS + organoids.” Protocols were included if they generated a CNS organoid through a novel technique. A second search was performed to identify viral pathogenesis studies using CNS organoids; Pubmed, Scopus, and EMBASE were searched using the terms “brain + virus + organoid” and “CNS + organoid + virus.” Manuscripts describing virus infection of CNS/brain organoids were included and paired with the organoid model type.

## 2. Spheroids vs. Organoids

We found, through the course of writing this review, that several manuscripts incorrectly described their models as brain organoids when they were actually spheroid aggregates of specific cell types [55,56]. Further, there were manuscripts incorrectly described as spheroids when they were actually brain organoids [45,57]. A spheroid culture is a 3D cell culture product where a single type of cell is grown in aggregates in a scaffold-free environment [58]. Multicellular spheroids can be made of two or more cell types grown together in aggregates or they can be derived through guided differentiation of PSCs [58]. Spheroids usually lack polarity and are unable to mimic the composition and functionality of tissues or organs [58]. While there are methods of generating specific cell types from pluripotent stem cells via guided differentiation, these models produce one or more cell types grown without a basement membrane and do not contain cells of multiple lineages [21]. Organoids are also sphere-shaped; however, they are 3D structures generated from PSCs or organ progenitor cells and are typically grown on a scaffold or basement membrane such as Matrigel [58]. Organoids, unlike spheroids, represent the cellular heterogeneity and physiology of organs and differentiate into cells of endo-, meso-, and ectodermal lineage whereas spheroids do not [58]. Recent work has defined organoids derived from guided differentiation as spheroids when they should be referred to as region-specific organoids since the resultant product differentiated into cells of multiple lineages [45,57]. Studies performing guided differentiation of pluripotent stem cells to produce spheroids should present data indicating that multiple cell lineages are absent.

## 3. Cell Lines Used for Organoid Generation

Choice of cell line used for generation of any organoid is integral for ensuring that the end product meets the requirements for generating or testing a hypothesis. hiPSCs are derived from primary blood monocyte cells, fibroblasts, epithelial cells and a variety of other cell types and can form all germ layers but cannot form extra-embryonic structures such as the placenta [59]. hESCs are derived from the inner cell mass of preimplantation embryos and can have restrictions for use to some researchers [59]. Regardless of source, stem cells should have a record of quality control and authentication to ensure pluripotency and genetics. hiPSCs should be chosen from lines that have been generated via the use of a non-integrating vectors such as Sendai virus or other episomal-type vector such that reprogramming vectors do not integrate with the host genome [60]. Cells should have a validated normal karyotype since reprogramming and passaging can compromise genetic integrity [60]. Finally, cell lines should have their pluripotency validated via phenotypic assays [60]. Usually, these quality control measures are performed by the vendor but should be repeated after gene editing, before cell banking, or when cultures exhibit unusual properties. 

Researchers have used a variety of hESCs and hiPSCs to generate brain organoids. The most widely used source for hiPSCs is WiCell, with the WA09 cells being popular for CO generation (Table 1). WiCell lines WA01, WA07, iPS (IMR90)-2, and ES03 are also commonly used (Table 1). hiPSC cell line cost varied from USD 495 to 1623 (Table 1). Many other cell lines were mentioned in the literature, but they were from research group biobanks and are not widely available to the public. While the purchase of stem cells can seem cost prohibitive, they can be passaged as long as they maintain a normal karyotype and pluripotency markers. While it is possible to use neural progenitor cells as a starting point for organoid generation, neural progenitor cells are not widely available from sufficient numbers of donors to make disease modeling possible.

Virus researchers typically present data from brain organoids derived from a single cell line, although some recent studies have derived brain organoids from two unique cell lines [55,61,62,63,64,65,66]. While dozens of organoids can be produced through a single generation protocol, the resultant organoids are basically clones of each other and donor-specific characteristics persist in unique cell lines. Thus, when designing an experiment, organoids should be derived from multiple cell lines. A recently published report showed that neuronal cell studies focused on disease modeling used five cell lines per study (three diseased, two control) or at least three cell lines for nondisease modeling [67]. However, in depth analysis of preliminary concepts requires substantial resources and time that is not justifiable for pilot studies, especially when generating organoids. Thus, preliminary data are often limited to two cell lines (control and diseased) [68,69,70].

**Table 1:** Commonly used cell types for CNS organoid generation. * price is for academic or non-profit institutions.

Cell Line	Cost USD	Organoids Generated	Company
WA01 (H1) [32,35,71]	USD 1250	Cerebral, Midbrain, Cortical, Fused MGE-cortical organoids	WiCell
WA09 (H9) [28,35,37,51,53,67,69,71,72,73,74,75,76]	USD 1250	Cerebral/ChP, Midbrain, Fused Dorsal-Ventral Cerebral, Vascularized Brain Organoid	WiCell
WA07 (H7) [77]	USD 1250	Neocortex	WiCell
Human Cord Blood CD34+ Cells (Cat #200-0000)	USD 634	Cerebral/ChP	STEMCELL Technologies
U87-MG [65]	USD 495	Cerebral	ATCC
GM00942, GM08330, GM00969 [78,79,80,81]	USD 650	Whole Brain organoids, Cerebral, Dorsally Patterned Forebrain Organoids	Coriell Institute *
BJ [82]	USD 495	Cerebral	ATCC
KYOU-DXR0109B Human IPS Cells [83]	USD 1623	Cerebral	ATCC
KhES-1 [34,48]	USD 244	Cerebral, Neocortex	RIKEN BioResource Research Center *
iPS(IMR90)-2 [84]	USD 1250	Whole Brain Organoid	WiCell
ES03 (HES-3) [32]	USD 1250	Fused Organoid	WiCell

## 4. Unguided Organoid Models

Several protocols for the generation of unguided brain organoids were found in Pubmed, Scopus and EMBASE. The most frequently mentioned protocol to create unguided brain organoids was by Lancaster et al. to create COs [21,24,25]. Most unguided brain organoids mature at about 2 months and can be kept for a year or more [30]. The longer that organoids are kept in culture, the later in fetal development they can represent, and organoids cultured after 250 days mimic post-natal brain development [30]. The unguided, Lancaster-based protocol has been performed with hePSCs, hiPSCs, and hiPSCs grown on mouse embryotic fibroblasts (MEFs) (Table 2) [85]. Stemcell Technologies offers a kit based on the Lancaster protocol that produces COs in roughly 54 days, which can be cultured for at least a year post maturation [30]. The estimated cost for the reagents used in the typical Lancaster-based protocol is about USD 5200, not including cells (Table 2). The kit available from Stemcell Technologies costs USD 359.00 with an additional USD 750 in plates and additives needed. Our search of the literature identified several manuscripts that used a modified version of the Lancaster method, although no acknowledgement to Lancaster et al. was made. We also found a manuscript that generated organoids from hiPSC derived from urine epithelial cells, which did not acknowledge Stemcell Technologies, who published this method of hiPSC generation in 2018 [86,87].

Several viral pathogenesis studies have been published using the unguided protocol, most likely due to the reproducibility of the Lancaster method and the numerous modifications available to it (Table 2) [25]. This protocol has been used to generate brain organoids to study Zika virus (ZIKV), Dengue virus (DENV), SARS-CoV-2, La Crosse encephalitis virus (LACV), measles virus (MeV), human cytomegalovirus (HCMV), and herpes simplex virus 1 (HSV-1). Two papers utilized COs to model ZIKV infection in vitro and were able to demonstrate that COs can exhibit characteristics of microcephaly [104,105]. This was a major advancement since ZIKV-related microcephaly can only be produced in mice by knocking out Type 1 interferon [102]. Dang et al. was also able to show that TLR3 was upregulated in ZIKV infected COs, which followed data obtained from human patients with acute ZIKV infection [103,105]. Numerous other papers also used COs to study ZIKV. Long et al. used COs to study how a ZIKV infection affects tubular matrices in the central endoplasmic reticulum, which validated other in vitro and animal research of ZIKV infection of endothelial cells [106,107,108,109]. Cavalcante et al. showed that ZIKV infected COs’ neuronal cells had reduced SOX^2+^ and increased Casp^3+^ expression which was also found in ZIKV infected chicken embryos [110,111]. Additionally, ZIKV infected COs exhibited irregular borders and cavities [110]. Cavalcante et al. were also able to show that betulinic acid (BA) can protect against ZIKV in COs [110]. 

Another group studied ZIKV oncolytic activity in COs co-cultured with CNS tumor cells and were able to showed that ZIKV diminished tumor growth, which was also found in mouse models and other organoid studies [93,112]. Another paper looked at saxitoxin (STX), which is produced by the cyanobacteria, *Raphidiopsis raciborskii*, and is common in Brazil [78]. This neurotoxin was shown to be harmful and doubled cell death in ZIKV infected brain organoids [78]. These findings have spurred further research into cyanobacterial and ZIKV infection [113,114]. Janssens et al. were able to show that ZIKV changes DNA methylation at particular loci in COs, which was also found in infants born with microcephaly [115,116,117]. Sacramento et al. used human brain organoids to reveal that sofosbuvir inhibits ZIKV replication [118]. These findings were also found in non-human primates and mice [119,120]. Lastly, Li et al. studied cortical organoid folding and growth in ZIKV and DENV infected organoids [74]. ZIKV infected organoids had major defects, while DENV infected organoids did not [74]. These findings support current knowledge that ZIKA causes fetal brain malformations in humans while dengue does not.

In addition to ZIKV, COs were used to study other viruses. Pellegrini et al. developed human brain organoids to show that SARS-CoV-2 can injure the choroid plexus [75]. Later reports in mice and human postmortem tissues validated their findings [64]. LACV, according to Winkler et al., diminished CO cell viability and that committed neurons were much more susceptible to LACV apoptosis than neural progenitors, which replicate data from humans, primates and rodents [83,121]. Using COs, Schultz et al. found that organoids derived from patients with Parkinson’s disease had increased pathology from Chikungunya virus (CHIKV) than organoids derived from normal patients [62]. This is intriguing since recent reports indicate a link between SARS CoV-2 and exacerbated Parkinson’s disease [122,123,124]. Qiao et al. used COs to study microglial activation by HSV-1 and was able to show that inflammatory factors were induced via infection which has also been shown in mice [125,126]. Wang et al. successfully infected COs with SARS-CoV-2 and were able to show that neurons that were co-cultured with astrocytes were infected at higher rates [92]. Brown et al. utilized COs to study HCMV and were able to see virus-induced alterations in morphology and saw hindered development of the organoids as a result of infection [127]. This mirrored changes that can occur during HCMV infection during pregnancy in humans [128]. Mathieu et al. studied MeV in COs and showed that MeV F mutations were linked with greater neuropathogenicity [76].

Other unguided approaches include the protocols by dos Reis et al. and Bodnar et al. Dos Reis et al. generated human brain organoids with microglia (MG-hBORGs) [94]. These researchers used neural progenitor cells (NPCs) to generate their organoids and co-cultured them with both primary and immortalized HIV-1 infected microglia [94]. This was important, as they tested a theorized mechanism for HIV to cause neuropathogenesis [129]. Bodnar et al. modified the protocol by Lancaster et al. and is notable because the researchers were able to control the ratio of microglia in the microglia-containing CO (MCO) because they kept the embryoid bodies in the same six well plates they used to generate the 3D spheres [79]. 

## 5. Guided Organoid Models

The majority of brain organoid generation methods found in the literature are guided methodologies. The main advantage of a guided methodology is control over region specificity through the use of inhibitors and patterning molecules [21]. There are guided methods for creating midbrain, dorsal and ventral cerebral, neocortex, forebrain, telencephalic, midbrain, and hypothalamus organoids (Table 1). Additionally, there are protocols for a fused dorsal-ventral CO and another for a fused human cortical (hCO) and human medial ganglionic eminence (MGE) organoid (hCO-hMGEO) [32,101].

Viral studies performed on these organoids were with SARS-CoV-2, ZIKV, and Japanese Encephalitis Virus (JEV) (Table 2). SARS-CoV-2 was modeled using dorsal forebrain organoids infected with pseudovirus [77]. The researchers were able to show that the SARS-CoV-2 pseudovirus was significantly co-localized with ACE2 compared to the control, which suggests that the virus was infecting the organoids via that receptor [77]. These findings were later validated in human postmortem tissues and mice [129]. Wang et al. cocultured pericyte-like cells (PLCs) with cortical organoids to create PLC-containing organoids (PCCOs) and were able to infect them with SARS-CoV-2. They were able to show that PCCOs are an acceptable model for studying SARS-CoV-2 in the human CNS [97]. McMahon et al. illustrated that SARS-CoV-2 targets glial and choroid plexus cells in cortical organoids which has also been shown for human and rodent models [64,130]. Another protocol looked at the effects of JEV on telencephalon organoids and demonstrated that JEV causes cell death in organoids but also that organoids can develop immunity to JEV, and in more mature organoids, there was an interferon response to JEV infection [89]. These findings validated rodent research, and were important since the mechanisms behind human neuroinflammation are not understood and limited data exist due to lack of human specimens [131].

Seven papers used guided brain organoids to model ZIKV infection. Three of these papers showed that CNS organoids are appropriate models for ZIKV microcephaly as they reflected pathology in humans and mice [50,66,96]. Watanabe et al. showed that ZIKV does infect NPCs and stunts organoid development [96]. Qian et al. used a patented spinning bioreactor (SpinΩ) to generate consistent forebrain organoids that were infected with ZIKV, which caused reduced organoid growth and size [50]. Xu et al. showed that ZIKV infected brain organoids modeled microcephaly since the virus caused the ventricular zone (VZ) layer to thin [66]. More mature organoids in this study experienced VZ and sub-ventricular zone disorganization, damage to the lumen and catastrophic cell death when infected with ZIKV [66]. The researchers were also able to prevent ZIKV damage in organoids through treatment with enoxactin [66]. Xu et al. used guided organoids to study ZIKV infection by co-culturing neural progenitor cells and primitive macrophage progenitors to create COs with precise concentrations of microglia [33]. This was useful in studying ZIKV in the brain because they were able to show that microglia prune synapses and phagocytize and respond to viral infection [33]. Xu et al. was able to use forebrain organoids to study how ZIKV is affected by small molecule inhibitors through screening [132]. Li et al. studied the how the niclosamide compound JMX0207 inhibits ZIKV infection in “mini-brain organoids” [133]. Another paper by the same research group also showed that methylene blue suppresses ZIKV infection in brain organoids [134].

## 6. Organoid Co-Culture to Address Model Limitations

CNS organoids lack sufficient microglia, monocytes, and vasculature which can make disease modeling difficult [29]. A recent advancement in brain organoid models was the co-culture with other cell types [29]. Brain organoids and cells have strict individual culture requirements; however, as long as the necessary components for each cell type are present, co-culture is a viable option for adding cell types to organoids to better replicate a whole system. 

Neuroinflammation is caused by activated microglia and invasion of CD4^+^ and CD8^+^ T-cells [135]. This occurs in response to injuries, infections, and genetic conditions. Brain organoids do not typically contain microglia sufficient for research, although a homemade microglia-containing cerebral model has been published [33]. Methodologies for microglia co-culture are used to model neuroinflammation on the brain. Methods include homemade organoid generation based on the Lancaster method and commercial kits [136,137]. These models show that microglia morphologies change in response to organoid injury, and they can migrate from the culture media into the organoid [33,136,137]. CD4^+^ T cells have been co-cultured with brain organoids to study the effects of necrotizing enterocolitis in the brain [138]. Using this model, Zhou et al. showed that gut derived CD4^+^ lymphocytes produced brain injury [138].

A major limitation for brain organoids is lack of vasculature, which results in central hypoxia and malnutrition when cultured over long periods of time, giving rise to a “necrotic core” [58]. Pham et al. devised a method to promote vascularization of COs by embedding organoids in Matrigel containing endothelial cells [139]. Song et al. achieved vascularization via tri-culture of neural progenitor cells, mesenchymal stem cells and endothelial cells [140]. With this method, organoids expressed several markers of the blood brain barrier (BBB) including ZO-1, GLUT1, BCRP and PGP [140]. Recent developments in co-differentiation and fusion have led to longer survival of organoids and better neural differentiation [71,141].

Viral studies utilizing co-culture methods with brain organoids are beginning to be reported. Some researchers utilized co-culture of specific cell types with their organoids. For unguided brain organoids, Abud et al. co-cultured the organoids with microglia-like cells (iMGLs) [142]. dos Reis et al. cultured their brain organoids with HIV-1 infected microglia [94]. For the guided organoid protocols, Xu et al. co-cultured COs with human cortical tissue, while Bershteyn et al. cultured NPCs with PMPs [33,95].

## 7. Conclusions

Clearly, there are many options for researching viral pathogenesis in brain organoids (Table 3). As Jacob et al. wrote: “Brain organoids offer a simple, accessible, and tractable human cell platform to investigate cellular susceptibility, disease mechanisms, and treatment strategies [55]”. Because brain organoids generated using unguided methods produce cell types of all lineages, they are best used for experimentation aimed at generating hypotheses or preliminary data. Unguided protocols are typically less expensive than guided protocols. Organoids generated via guided methods produce specific cell types reflecting specific regions of the brain and are useful for experimentation aimed at testing hypotheses. Most viral pathogenesis studies used the Lancaster method or a modification of the Lancaster method. Regardless of methodology used to generate brain organoids, further studies using human patients are necessary to validate findings. 

There is a wide range of costs for generating any type of brain organoid. Media and supplements have limited shelf life while growth factors and patterning factors can cost thousands of dollars per unit, which can make research using these models cost prohibitive especially for generating preliminary data. Kits available from Stemcell Technologies provide a less expensive and consistent platform for guided and unguided organoid generation that can make organoid methodology accessible to more researchers. 

Although there are limitations to using brain organoids, many can be addressed through the use of specific patterning factors and co-cultures such that animal models can be eliminated or greatly reduced. With ongoing advancements in cell culture and bioengineering, limitations for brain organoid models or shrinking and data production are resulting in better understanding and treatment of viral infections of the CNS.

There are numerous guided and unguided methods for generating organoids representing multiple regions of the brain, with several types of modifications for fine-tuning the model to a researcher’s specifications. Organoid models of the CNS can serve as a platform for characterization and mechanistic studies that can reduce or eliminate the use of animals, especially for viruses that only cause disease in the human CNS. Organoid generation can be costly and time consuming, and choosing the correct methodology is paramount for ensuring experimental aims are met. However, regardless of methodology used, viral studies using brain organoids have advanced our understanding of human neuropathogenesis. 

## Figures and Tables

**Table 2:** Guided and unguided brain organoid models. Expense was calculated by summing the costs of one unit of all the reagents listed in the protocols in USD. The time column specifies the number of days to generate and organoid starting with hiPSC.

Organoid Type	Protocol	Cost USD	Modifications	Time	Virus	Kit
**Unguided**						
CO [23,27,28,54,55,56,57,58,59,60,61,62,63,64,65,66,67,68,69,70,71,88,89,90]	Lancaster et al., 2014 [24,25]	USD 5219.23 Kit = USD 1100	Co-culture with iMGL; feeder cells can be used [85,91]; Co-cultured neurons with astrocytes [92]; Co-cultured with CNS tumor cells [93]	2+ months	ZIKV, DENV, SARS-CoV2, LACV, HSV-1, CHIKV, HCMV, MeV	STEMCELL Technologies
CO	Bodnar et al., 2021 [79]	USD 3611.57	NR	35–80 days	NR	−
CO/Chp	Pellegrini et al., 2020 [72]	USD 1127.15	NR	54 days	NR	STEMCELL Technologies
MG-hBORGs	dos Reis et al., 2020 [94]	USD 2142.67	Co-culture with HIV-1 infected microglia [94]	99–265 days	HIV-1	−
**Guided**						
Midbrain-like (hMLO)	Jo et al., 2016, Monzel et al., 2017 [35,36]	USD 5731.23	SMAD inhibition [35] and *Wnt* pathway activation [35,36], SHH pathway activation [36]	1–2 months	NR	−
CO/Neocortex/Cortical/Dorsally Patterned [48,81,95,96]	Kadoshima et al., 2013 [34]	USD 2314.18	Rho kinase [34,95], *Wnt* and TGFβ inhibitors [34,48,81,95]; Co-cultured cerebral organoids with human cortical tissue [95]; FGF19 [48]; Co-cultured with pericyte-like cells [97]	3 months	SARS-CoV2, ZIKV	−
Telencephalic Organoids	Mariani et al., 2015 [22,49,98]	USD 3846.86 Kit = USD 1100	NR	115 days	NR	−
Forebrain, Midbrain, & Hypothalamus	Qian et al., 2018 [50,99]	USD 4413.81	SMAD inhibition [50]	71–80 days	ZIKV	−
Telencephalon Cortical	Zhang et al., 2018 [89]	USD 3038.49	NR	24–91 days	JEV, ZIKV	−
CO	Lindborg et al., 2016 [54]	USD 3234.65	Can use feeder cells [54]	28 days	NR	−
CO	Xu et al., 2021 [33]	USD 5460.08	Co-culture of neural progenitor cells (NPCS) with primitive macrophage progenitors (PMP); dual SMAD inhibition [33]	35 days	ZIKV	−
hCO	Cakir et al., 2020 [71]	USD 3767.62	NR	1–3 months	NR	−
Ventral and Dorsal Forebrain Organoid	Birey et al., 2017 [100]	USD 3844.56	SMAD inhibition, *Wnt* inhibition, SHH agonist [100]	43 days	SARS-CoV2	STEMCELL Technologies
Fused dorsal-ventral CO	Bagley et al., 2017 [101]	USD 4622.15	Ventral drug treatment (IWP2 + SAG) and dorsal treatment (CycA) EBs that were embedded together and eventually fused; feeder cells can be used [101]	100 days	NR	−
Human Cortical Spheroids in Feeder Free Conditions (hCS-FF)/ Forebrain Organoid	Yoon et al., 2019 [45]	USD 3825.68	SMAD and *Wnt* inhibition, EGF and FGF2 growth factors; can modify patterning molecules used to create forebrain organoids	100+ days	ZIKV [102,103]	−
Fused MGE-hfMCO and Fused hMGEO and hCO	Xiang et al., 2017 [32]	USD 2573.77	Fused; SMAD inhibition and SHH activation, *Wnt* signaling [32]	2+ months	NR	−

**Table 3:** Comparison of organoid models for their use in experimentation.

Purpose	Preliminary Data, Hypothesis Generating, Hypothesis Testing	Preliminary Data, Hypothesis Generating, Hypothesis Testing	Hypothesis Testing	Hypothesis Testing	Hypothesis Testing
Anatomy	Forebrain	Cerebrum	Region Specific i.e., midbrain, telencephalic etc.	Forebrain with BBB or vasculature	Fused organoids
Methodology	Guided	Unguided	Guided	Guided	Guided/ Unguided
Reagents	STEMdiff™ Dorsal or Ventral Forebrain Organoid Differentiation Kits	STEMdiff™ Cerebral Organoid Kit	Homemade	Homemade	Homemade but may use kits to generate forebrain portions
Cost	$	$	$$	$$$	$$$$
Time	++	++	++	++	+++
Reasoning	Dorsal kit generates dorsal pallium, ventral kit generates ventral sub pallium; inexpensive, technical support available	All cell types are present, Whole cerebrum represented; inexpensive, technical support available	Focuses on specific neuron/glia type.	Useful for understanding vasculature in viral pathogenesis in CNS.	Useful for modeling interactions between specific brain regions.
Modifications		Add choroid plexus; co-culture with microglia	Patterning factors can be added to promote cell types of interest	Patterning factors can be added to promote cell types of interest	Patterning factors can be added to promote cell types of interest
Difficulty	+	+	+++	++++	+++++

+ Relative added protocol difficultly or time. More symbols means the protocol is either more difficult or takes more time. $ Relative cost of the protocol. More symbols indicates the protocol is more expensive (See Table 2 for estimated costs).
